# Molecular Profiling of *SYT-SSX* Fusion Transcripts for Enhanced Diagnosis of Synovial Sarcomas

**DOI:** 10.3390/jpm15100455

**Published:** 2025-09-29

**Authors:** Sara Louati, Kaoutar Bentayebi, Ibtissam Saad, Yvonne Gloor, Nadia Senhaji, Abdelmajid Elmrini, Lahcen Belyamani, Rachid Eljaoudi, Marc Ansari, Sanae Bennis, Youssef Daali

**Affiliations:** 1 Medical Biotechnology Laboratory, Medical and Pharmacy School, Mohammed V University of Rabat, Rabat BP 10000, Morocco; s.louati@um5r.ac.ma (S.L.); k.bentayebi@um5r.ac.ma (K.B.); r.eljaoudi@um5r.ac.ma (R.E.); 2 Mohammed VI University of Sciences and Health (UM6SS), Casablanca 20000, Morocco; isaad@um6ss.ma (I.S.);; 3 Mohammed VI Center for Research and Innovation (CM6RI), Rabat 10112, Morocco; 4 CANSEARCH Research Platform for Pediatric Oncology and Hematology, Department of Pediatrics, Gynecology and Obstetrics, Faculty of Medicine, University of Geneva, 1205 Geneva, Switzerland; yvonne.gloor@unige.ch (Y.G.); marc.ansari@hcuge.ch (M.A.); 5 Department of Biology, Faculty of Sciences, Moulay Ismail University of Meknes, Meknes 50000, Morocco; n.senhaji@umi.ac.ma; 6 Department of Orthopaedic Surgery B4, CHU Hassan II Hospital, University of Sidi Mohammed Ben Abdellah, Fez 30000, Morocco; 7 Emergency Department, Military Hospital Mohammed V, Rabat 10000, Morocco; 8 Pharmacology and Toxicology Department, Medical and Pharmacy School, Mohammed V University in Rabat, Rabat 10000, Morocco; 9 Division of Pediatric Oncology and Hematology, Department of Women, Child, and Adolescent, University Hospital of Geneva, 1205 Geneva, Switzerland; 10 Laboratory of Biomedical and Translational Research, Faculty of Medicine, Pharmacy and Dental Medicine of Fez, Sidi Mohamed Ben Abdellah University, Fez 30000, Morocco; sanae.bennis@usmba.ac.ma; 11 Division of Clinical Pharmacology and Toxicology, Department of Anesthesiology, Pharmacology, Intensive Care and Emergency Medicine, Geneva University Hospitals, 1205 Geneva, Switzerland; 12 Faculty of Medicine, University of Geneva, 1205 Geneva, Switzerland; 13 School of Pharmaceutical Sciences, University of Geneva, 1205 Geneva, Switzerland

**Keywords:** synovial sarcoma, *SYT-SSX* fusion transcripts, molecular diagnostics

## Abstract

**Background/Objectives**: Synovial sarcoma (SS) is an aggressive soft-tissue tumor characterized by the chromosomal translocation t(X;18) (p11.2;q11.2), most commonly involving the fusion of the *SYT* gene on chromosome 18 with the *SSX1* or *SSX2* genes on chromosome X. This study aims to explore the clinicopathological and molecular characteristics of synovial sarcoma in a cohort of Moroccan patients. **Methods**: We analyzed 48 cases of synovial sarcoma using formalin-fixed, paraffin-embedded (FFPE) tissue samples. Histological grading was performed according to the FNCLCC system. Immunohistochemical staining was employed to detect cytokeratin (CK) and epithelial membrane antigen (EMA). Molecular analysis included fluorescence in situ hybridization (FISH) to identify *SS18* gene rearrangements and reverse transcription–polymerase chain reaction (RT-PCR) to detect *SYT-SSX* fusion transcripts. **Results**: Among the cohort, 56% of cases showed *SS18* gene rearrangements via FISH, while RT-PCR confirmed the presence of *SS18-SSX1* and *SS18-SSX2* transcripts in 60% and 32% of cases, respectively. The remainder was classified as undifferentiated sarcoma. Notably, no significant associations were observed between *SYT-SSX* fusion type and clinicopathological features. **Conclusions**: These findings underscore the importance of integrating molecular techniques for precise diagnosis in synovial sarcoma. The results align with global patterns, emphasizing the necessity for molecular testing to enhance diagnostic accuracy and informing potential therapeutic advancements.

## 1. Introduction

Synovial sarcomas are aggressive tumors that account for 7–10% of all soft-tissue malignancies, primarily affecting the extremities of young adults. These tumors present significant diagnostic and therapeutic challenges due to their invasive nature and variable clinical presentations [1].

Histologically, synovial sarcoma is a monomorphic spindle cell sarcoma with varying degrees of epithelial differentiation, and it is divided into two major morphological subtypes: monophasic and biphasic [2]. Monophasic synovial sarcoma, which constitutes 60–75% of cases [3], is composed predominantly of short fascicles of spindle cells, often with occasional epithelioid areas. In contrast, biphasic synovial sarcomas are characterized by the presence of both spindle cells and epithelial cells, typically arranged in glandular structures [4]. A poorly differentiated pattern, defined by the presence of highly atypical, mitotically active round or spindle cells, has also been recognized, but it is rare [5].

In addition to clinical features and microscopic examination, immunohistochemical analysis is another key indicator in diagnosing synovial sarcomas. More than 90% of synovial sarcoma cases, regardless of subtype, show focal expression of epithelial markers such as cytokeratins (CK) and epithelial membrane antigen (EMA). These markers typically display a patchy staining pattern in spindle cell components and more uniform staining in epithelial regions, and they are used complementarily to ensure diagnostic accuracy [6,7].

This sarcoma subtype is defined by the pathognomonic t(X;18) chromosomal translocation, which results in the formation of the *SS18-SSX* fusion oncoprotein [8]. Present in over 95% of synovial sarcoma cases across all morphological subtypes, this translocation is highly specific to synovial sarcoma and absent in other tumors [9]. While diagnosis is primarily based on histomorphology and immunophenotyping, molecular detection of the *SS18-SSX* fusion is increasingly seen as indispensable in distinguishing SS from other spindle cell sarcomas and carcinomas.

Most cases of synovial sarcoma exhibit a fusion between the *SS18* gene and either the *SSX1* or the *SSX2* gene, creating *SS18-SSX1* or *SS18-SSX2* gene rearrangements. Approximately two-thirds of the cases harbor the *SS18-SSX1* fusion, while the remainder carry the *SS18-SSX2* fusion [10].

Detecting these *SYT-SSX* fusion transcripts serves as a definitive diagnostic marker, especially when more conventional methods, such as FISH, are inconclusive.

Beyond diagnosis, the type of *SYT-SSX* fusion is closely linked to the tumor’s histological subtype, biological behavior, and prognosis. Some studies have demonstrated that *SYT-SSX1* is more inclined to locate in the epithelial components of biphasic synovial sarcoma, whereas monophasic synovial sarcoma harbors *SYT-SSX2*. Prognosis also varies, with patients carrying the *SYT-SSX2* fusion generally experiencing better metastasis-free survival compared to those with *SYT-SSX1* [11] and demonstrating the highest survival rates at both five and ten years, with Male and black race were independently associated with even worse survival in the monophasic subtype [12].

Since synovial sarcoma exhibits genomic stability with few somatic mutations aside from the *SS18-SSX* translocation, the fusion oncoprotein SS18-SSX is considered the key oncogenic driver, disrupting differentiation through widespread epigenetic dysregulation [13,14,15]. Despite this, over a quarter of synovial sarcoma patients die from metastatic disease within five years of diagnosis, yet the precise pathogenic mechanisms remain unclear.

Therefore, there is a pressing need to further explore the pathogenesis, invasion, metastasis, and therapeutic strategies for synovial sarcoma, particularly molecular-targeted treatments. Recent research suggests that the *SYT-SSX* fusion gene may be regulated by various signaling pathways, microRNAs, and other molecules, contributing to stem cell-like characteristics or promoting epithelial–mesenchymal transition, ultimately leading to increased invasion and metastasis [11].

Furthermore, specific *SYT-SSX* fusion transcripts represent promising molecular targets for novel therapies. For instance, interactions between fusion transcripts and cell cycle regulators (such as *SS18-SSX1*) have been observed, making these pathways potential targets for treatment. Just as imatinib mesylate has been successful in treating the translocation-associated tumor chronic myeloid leukemia, developing drugs that effectively target the *SS18-SSX* fusion could provide new hope for synovial sarcoma patients [16].

Currently, routine characterization of *SS18-SSX1* and *SS18-SSX2* fusion transcripts is not common, leading to limited understanding of how their detection may affect diagnosis and prognosis, particularly in Moroccan patients, who may have a distinct genetic profile. To address this gap, our study reports on the histologic, immunohistochemical, and cytogenetic profiles of synovial sarcomas, along with the characteristics of *SS18-SSX* chimeric fusion genes, within a cohort of Moroccan patients.

## 2. Materials and Methods

### 2.1. Study Design and Patients

Following ethical approval from the institutional review board at University Sidi Mohamed Ben Abdelah in Fez, we conducted a retrospective analysis utilizing formalin-fixed, paraffin-embedded (FFPE) tissue samples from the Pathology Department. Our study comprised 48 cases of synovial sarcoma, with demographic details such as age and gender provided in the Results section. Each case was meticulously evaluated by board-certified pathologists, ensuring diagnostic accuracy through independent review.

All tissue samples underwent staining with hematoxylin and eosin (H & E) to assess tumor adequacy, defined as having at least 100 viable tumor cells for assay interpretation. Each staining run included prequalified human benign tissues, such as normal muscle and adipose tissue, serving as positive and negative controls to validate the specificity and accuracy of the staining.

### 2.2. Immunohistochemical Analysis

Immunohistochemistry (IHC) was performed using EMA (E-29, Dako) and CK (AE1/AE3, Dako, Glostrup, Denmark) antibodies, utilizing the LSAB kit (DakoPatts^®®^, Copenhagen, Denmark) on an automated immunostainer. Heat-induced epitope retrieval (HIER) was conducted prior to staining, with 3,3′-diaminobenzidine tetrahydrochloride (DAB) serving as the chromogen. Both positive and negative controls were included to ensure assay reliability. Results were considered positive when more than 10% cytoplasmic staining was observed in tumor cells.

### 2.3. Fluorescence in Situ Hybridization

FISH analysis was carried out to detect the *SS18* gene rearrangement in synovial sarcomas using the Vysis SS18 Break Apart FISH Probe kit. This kit includes two LSI SS18 probes located at 18q11.2. The FISH was performed on 3.5 μm-thick sections according to the manufacturer’s recommendations. In normal cells lacking t(18q11.2), a two-fused signal pattern (yellow) indicates two intact copies of the *SS18* gene. In abnormal cells with t(18q11.2), a split signal pattern (one green and one red signal) is observed. A minimum of 100 non-overlapping tumor cells per sample were evaluated to assess the presence of fused or split green and red signals. A positive result was defined as more than 20% of cells exhibiting split signals.

### 2.4. RNA Extraction and RT-PCR Assay

Prior to RT-PCR, paraffin-embedded samples were sectioned and scraped into Eppendorf tubes. RNA was extracted using the RNeasy^®^ FFPE Kit (QIAGENT, Germantown, MD, USA) according to the manufacturer’s instructions. Total RNA was quantified using the Qubit^®^ 3.0 Fluorometer (Thermo Fisher Scientific). RNA was then converted to cDNA using the Prime Script™ RT Reagent TAKARA, Richmond, CA, USA. The integrity of RNA was evaluated by PCR for the β2 microglobulin gene (B2M) using forward (5′-TGA CTT TGT CAC AGC CCA AGA TA-3′) and reverse (5′-AAT CCA AAT GCG GCA TCT TC-3′) primers. Samples with insufficient RNA quality were excluded from further analysis.

RT-PCR was performed on positive FISH cases to characterize the most common fusion transcripts: *SS18-SSX1* and *SS18-SSX2*. Thermal cycling conditions were: 7 min at 95 °C, followed by 40 cycles of 30 s at 94 °C, 30 s at 62 °C, and 30 s at 72 °C. Amplicons were visualized on a 2% agarose gel stained with Cybersafe™.

### 2.5. Sequencing Analysis

PCR products corresponding to fusion transcripts were extracted and purified from the gel using the DNA Clean & Concentrator™-5 Kit (Zymo Research, Irvine, CA, USA). Sequencing PCR was performed with a 10 μL reaction volume containing 1 μL of Big Dye Terminator V3.1, 2 μL of SS primers, 1.5 μL of sequencing buffer, and 2 μL of purified PCR products, under the following conditions: 25 cycles of 10 s at 95 °C and 5 s at 50 °C. The sequencing product was purified using the BigDye^®^ Xterminator™ Kit, (Life Technologies Corporation, Austin, TX, USA) and sequenced by the Sanger method. Electropherograms were edited, and breakpoints were analyzed. Bioinformatics tools and the BLAST+ 2.8.1 software were used to align the sequences with Homo sapiens synovial sarcoma (SSX) [NCBI reference sequence: NG_012528.1]. The nucleotide sequences were also aligned with Homo sapiens chromosome 18, primary assembly GRCh37.p5 [NCBI reference sequence: NC_000018.9] for the *SS18* region.

### 2.6. Statistical Analysis

The statistical analyses for this study were conducted using Python 3.12.4 within the Anaconda environment. Descriptive statistics provided demographic insights into the cohort, including age and gender distribution. Fisher’s Exact Test was used to evaluate associations between *SYT-SSX* fusion types and clinicopathological variables like tumor location, sex, and morphology, while odds ratios (OR) quantified the strength of these associations.

For continuous variables, independent *t*-tests compared tumor grades across sex and fusion types, assessing whether differences in mean grades were statistically significant. The *t*-test outputs included *p*-values to determine statistical significance and Cohen’s d for calculating the effect size, providing a measure of the magnitude of these differences beyond statistical significance.

To visualize relationships and patterns in the data, Multidimensional Scaling (MDS) was applied, particularly to examine the relationships between fusion type, tumor grade, and sex, providing a two-dimensional representation of similarities between cases. Additionally, heatmaps were generated to depict the expression levels of CK and EMA markers, stratified by tumor morphology and sex, offering a quick visual interpretation of the immunohistochemical data.

All statistical tests and visualizations were performed using Python’s SciPy for statistical tests, Pandas 2.2.2 for data manipulation and structuring, and Seaborn 0.13.2 and Matplotlib 3.9.2 for graphical representations. This integrated approach allowed for a comprehensive examination of the molecular and clinical characteristics of synovial sarcoma in this Moroccan cohort. The use of these advanced analytical techniques enabled the study to thoroughly explore the associations between *SYT-SSX* fusion types and clinicopathological features, while also providing insights into marker expression patterns that may influence tumor behaviour and prognosis.

## 3. Results

### 3.1. Clinical and Histological Features

**Figure 1:**
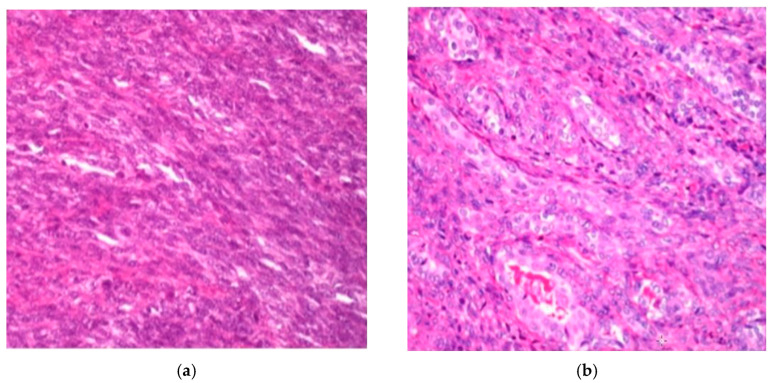
H&E staining (×250): (**a**) diffuse proliferation of spindle cells in monophasic synovial sarcoma; (**b**) and biphasic synovial sarcoma.

Histopathologic analysis was performed according to the 2013 WHO classification criteria, focusing on morphological characteristics. The cellular composition revealed two primary forms: monophasic spindle cell synovial sarcoma and biphasic spindle cell and epithelial synovial sarcoma (Figure 1).

In the appendices, Supplementary Table S1 summarize the features of our 48 cases.

**Figure 2:**
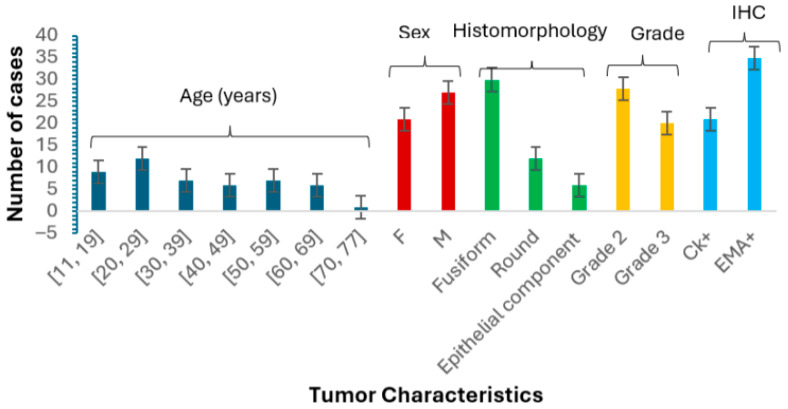
This bar graph illustrates the distribution of synovial sarcoma cases based on age, sex (F = female, M = male), histomorphology, tumor grade, and IHC markers. The chart highlights that males constitute 56% of the cohort, with the highest representation in the 20–29 age group (25%) and a notable presence of paediatric cases represented in the age category 11–19 (19%). Histomorphologically, 62.5% of tumors were fusiform, 25% were round, and 12.5% were epithelial. The majority of tumors were grade 2 (58%), while 42% were grade 3. Immunohistochemical analysis revealed EMA positivity in 35 cases and CK positivity in 21 cases. Error bars represent the standard error of the mean.

The analysis (Figure 2) indicated a slight male predominance, with males comprising 56% of the cohort. The most represented age group was 20 to 29 years (25%), followed by a significant paediatric population. Notably, only one sample was obtained from patients aged 70 to 77. Tumor location analysis showed that 60% of cases originated from the lower limbs, while others were found in the central axis and upper limbs. Histologically, 62.5% of tumors exhibited a fusiform morphology, 25% were round, and 12.5% demonstrated an epithelial component. Regarding tumor grade, 58% were classified as grade 2 and 42% as grade 3. Immunohistochemical analysis revealed that 35 cases tested positive for EMA, while 21 cases were positive for CK, indicating significant expression of these markers in the studied population.

### 3.2. Immunohistochemistry Analysis

**Figure 3:**
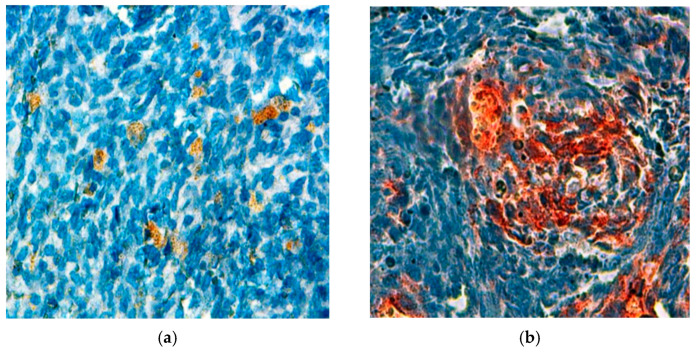
Cytoplasmic expression of antibodies observed in a synovial sarcoma (×400): (**a**) anti-CK; (**b**) anti-EMA.

Immunohistochemistry is a fundamental tool in the diagnosis of synovial sarcoma, allowing pathologists to distinguish between different tumor types based on specific marker expression. In our study, the staining patterns revealed that 56.25% (21 out of 48) of the cases exhibited positive staining for CK, a marker indicative of epithelial differentiation (Figure 3a). Additionally, a significant 72.9% (35 out of 48) of cases tested positive for EMA, further supporting the diagnosis of synovial sarcoma (Figure 3b). The presence of these markers highlights the importance of epithelial characteristics in synovial sarcoma, as they not only aid in the accurate classification of tumors but also provide insights into the biological behaviour of the disease. The substantial positivity for EMA suggests a strong epithelial component, which may have implications for prognosis and treatment strategies. Given these findings, incorporating immunohistochemical analysis into routine diagnostic practices is essential for refining the understanding of synovial sarcoma and ensuring precise therapeutic interventions.

### 3.3. Detection of the Presence of the Rearrangement by FISH

**Figure 4:**
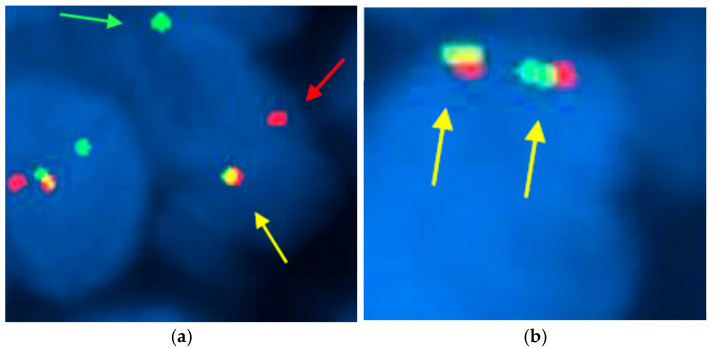
FISH technique using the Vysis SS18 break-apart probe in synovial sarcoma. (×1000): (**a**) In patient number 3, two break-apart red and green signals (red and green arrowheads) and one fused signal (yellow arrowhead) are observed, indicative of rearrangement in the *SS18* gene; (**b**) In patient number 27, two fused signals (yellow arrowhead) are observed, thus demonstrating no *SS18* gene rearrangement. The presence of separated signals in at least 20% of the nuclei indicates a positive result for the chromosomal alteration associated with this malignancy. This result confirms the presence of the chromosomal alteration associated with synovial sarcoma, highlighting the utility of FISH in diagnosing this malignancy.

FISH assay was conducted to assess the presence or absence of chromosomal rearrangements in the *SS18* gene, which is crucial for the diagnosis of synovial sarcoma (Figure 4). Our results showed that 56% (27 out of 48) of the cases exhibited chromosomal rearrangements, confirming the genetic alteration’s significance in this cohort. However, 6% (3 out of 48) of the samples were classified as uninterpretable due to factors such as weak fluorescent signals, insufficient tumor tissue, necrosis, or non-compliance with fixation protocols. Figure 4 presents an illustrative case of synovial sarcoma, highlighting the *SS18* gene rearrangement characterized by the distinct separation of red and green fluorescent signals in at least 20% of the nuclei. This clear visualization underscores the importance of FISH in accurately diagnosing synovial sarcoma and understanding its underlying genetic mechanisms.

### 3.4. Detection of SS18-SSX Fusion Transcripts by RT-PCR and Sequencing Analysis

The analysis of *SS18-SSX* fusion transcripts was conducted using RT-PCR on 27 tumors diagnosed as synovial sarcomas and confirmed by FISH. Two cases were excluded due to consistently negative amplification traces for the control β-2M, likely from insufficient mRNA extraction or degradation (Supplementary Figure S1). The results revealed *SS18-SSX1* and *SS18-SSX2* fusion transcripts in 60% (15 out of 25) and 32% (8 out of 25) of cases, respectively, while 8% (2 out of 25) showed no detectable fusion transcripts (Supplementary Figure S2). Sequencing confirmed the presence of the fusion transcripts corresponding to *SS18-SSX1* (Supplementary Figure S3) and *SS18-SSX2*.

### 3.5. Statistical Results

#### 3.5.1. Statistical Analysis of Clinicopathological Associations in Synovial Sarcoma: Fusion Type, Tumor Grade, and Sex

A comprehensive statistical analysis was conducted to examine the associations between *SYT-SSX* fusion types and various clinicopathological characteristics in synovial sarcoma cases. Fisher’s Exact Test was used to evaluate potential relationships between fusion type and factors such as age, sex, tumor location, and morphology. The analysis revealed no statistically significant differences between fusion types and clinicopathological features. The odds ratio for age (OR = 0.67) indicated that patients with *SYT-SSX1* fusion were 33% less likely to be under 30, though this was not statistically significant (*p* = 0.685). Similarly, the odds ratio for sex (OR = 1.14) suggested a slight male predominance in *SYT-SSX1* cases, but this association was not statistically significant (*p* = 1.00). For tumor location, the odds ratio of 4.8 pointed to a trend where *SYT-SSX1* fusion was associated with lower limb tumors, but the *p*-value (0.27) indicated that this result may be due to random variation.

*T*-tests were conducted to assess differences in tumor grades between sexes and fusion types, with no significant findings. The t-statistic for sex was 0.70 (*p* = 0.49), suggesting no grade difference between male and female patients. Similarly, no significant differences in grades were observed between fusion types, with *p*-values of 0.92 for *SYT-SSX1*, 0.68 for *SYT-SSX2*, and 0.78 for Unknown. Cohen’s d values indicated small effect sizes, with negligible practical differences between these groups. These results suggest that sex and fusion type do not significantly influence tumor grade or clinical outcomes in this cohort.

#### 3.5.2. Analyzing Tumor Location Patterns in Relation to *SYT-SSX* Fusion Types

**Figure 5:**
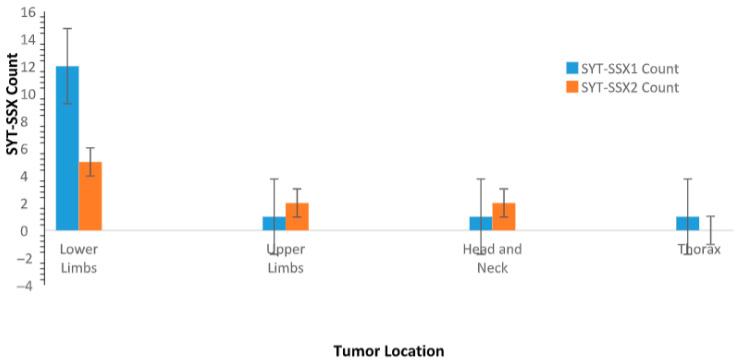
This bar graph presents the distribution of tumor locations (lower limbs, upper limbs, thorax, and head and neck) for patients with *SYT-SSX1* and *SYT-SSX2* fusion types. *SYT-SSX1* fusion type is more common in tumors located in the lower limbs, while *SYT-SSX2* fusion type exhibits a more even distribution across various anatomical locations. Error bars represent the standard error of the mean (SEM) for each group, indicating variability within the sample.

The distribution of tumor locations for patients with different *SYT-SSX* fusion types shows that *SYT-SSX1* is predominantly associated with tumors in the lower limbs (12 cases), with fewer cases in the upper limbs (1 case), thorax (1 case), and head and neck (1 case). On the other hand, *SYT-SSX2* shows a more even distribution, with 5 tumors located in the lower limbs, 2 in the upper limbs, and 2 in the head and neck. There are no *SYT-SSX2* cases observed in the thorax (Figure 5).

#### 3.5.3. Differential Expression of EMA and CK Markers by Tumor Morphology and Patient Sex

**Figure 6:**
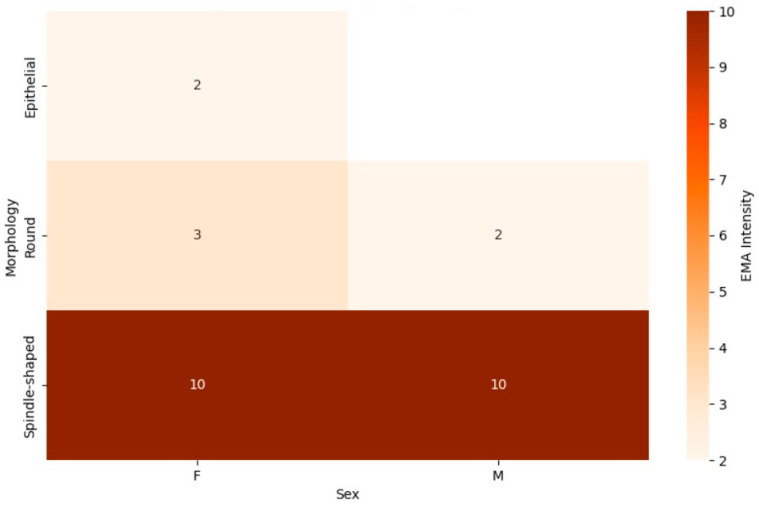
This combined heatmap illustrates the expression of the epithelial membrane antigen (EMA) marker in synovial sarcoma patients, categorized by tumor morphology (spindle-shaped, round, and epithelial) and sex (male and female). The color intensity represents the proportion of patients with positive EMA expression, with darker shades indicating higher positivity, allowing for comparisons across morphological subtypes and highlighting potential sex-specific variations.

The EMA marker heatmap (Figure 6) revealed distinct patterns of marker expression across different tumor morphologies and sexes. Among patients with spindle-shaped tumors, EMA expression was observed more frequently in female patients, with a higher proportion of females showing positive EMA staining compared to their male counterparts. Male patients with spindle-shaped tumors exhibited lower EMA positivity, indicating a potential sex-specific difference in EMA expression within this morphological subtype. Similarly, for round cell tumors, EMA positivity was predominantly seen in female patients, with a notable reduction in male patients. However, epithelial tumors exhibited consistently high EMA expression in both sexes, suggesting that this morphology may be more strongly associated with EMA positivity, regardless of sex.

**Figure 7:**
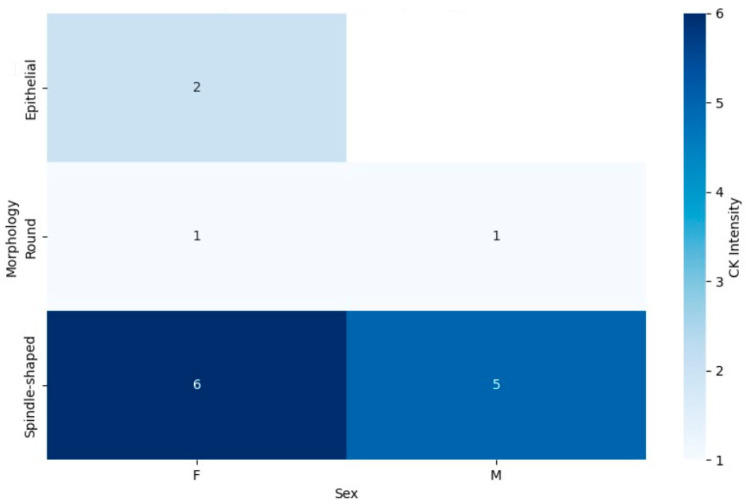
This combined heatmap illustrates the expression of CK marker in synovial sarcoma patients, categorized by tumor morphology (spindle-shaped, round, and epithelial) and sex (male and female). The color gradients represent the proportion of patients with CK positivity, with darker shades indicating greater expression. The distribution across tumor morphologies and sexes offers insights into potential diagnostic or prognostic implications related to marker expression patterns.

In contrast, the CK marker heatmap (Figure 7) demonstrated different trends in marker expression. Spindle-shaped tumors showed a relatively balanced CK expression between male and female patients, with a slight increase in positivity observed in males. For round cell tumors, CK expression was generally low in both sexes, with minimal variation between male and female patients. However, epithelial tumors once again displayed high levels of CK expression across both sexes, similar to the patterns observed in EMA expression. This suggests that epithelial morphology in synovial sarcoma may be closely associated with both CK and EMA marker positivity, indicating a more consistent phenotype across sexes.

#### 3.5.4. Multidimensional Scaling Analysis of *SYT-SSX* Fusion Types by Tumor Grade and Sex

**Figure 8:**
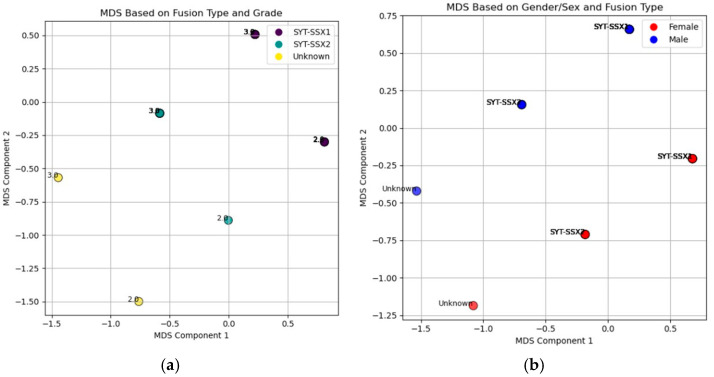
MDS plots illustrating the relationships between key clinical features in synovial sarcoma patients: (**a**) represents the relationship between fusion type (*SYT-SSX1*, *SYT-SSX2*, and Unknown) and tumor grade (Grade 2 and Grade 3). The color of each point corresponds to the patient fusion type, and the location of the points in two-dimensional space reflects the similarity or dissimilarity between patients based on both fusion type and tumor grade. This reduction into two dimensions allows for visualization of potential clustering patterns based on these variables; (**b**) displays the relationship between sex (male and female) and fusion type. The color of each point represents the patient’s sex, while different shapes denote the fusion types. The position of each point indicates the similarity or dissimilarity between patients in terms of both sex and fusion type, enabling a visual exploration of any potential clustering or grouping patterns in the data.

The Multidimensional Scaling (MDS) analysis revealed patterns in the relationships between clinical and molecular features of synovial sarcoma (Figure 8). In one plot (Figure 8a), the distribution of *SYT-SSX* fusion types across tumor grades (2 and 3) indicated no clear clustering, suggesting that tumor grade does not strongly differentiate *SYT-SSX1* from *SYT-SSX2* fusion types. Similarly, the MDS plot analyzing sex and fusion type (Figure 8b) showed no distinct separation between male and female patients, indicating limited influence of sex on fusion type distribution. These findings highlight the genetic and morphological complexity of synovial sarcoma.

## 4. Discussion

This study provides a comprehensive analysis of synovial sarcoma in a Moroccan cohort, revealing critical insights into its clinical and molecular characteristics while highlighting similarities and discrepancies with global data. Approximately 58.33% of cases occurred in individuals aged 11 to 39, with a slight male predominance (sex ratio of 1.3). This demographic pattern aligns with international data, confirming that synovial sarcoma predominantly affects adolescents and young adults without a significant gender bias. Additionally, the predominance of tumors located in the lower extremities, particularly near the knee, reaffirms established patterns observed globally [17,18].

The interconnections among *SYT-SSX* fusion types, tumor location, morphology, and sex were crucial in our analysis. We observed that tumors with the *SYT-SSX1* fusion type predominantly manifest in the lower limbs, supported by an odds ratio of 4.8. However, the lack of statistical significance (*p*-value = 0.27) suggests that this association may be due to random variation rather than a clinically meaningful relationship. Consequently, tumor location alone does not serve as a definitive distinguishing factor between *SYT-SSX1* and *SYT-SSX2*, emphasizing the need for larger studies to validate these observations. This finding aligns with research by [19] who reported similar patterns in tumor localization but without definitive statistical backing.

Our molecular analysis revealed that 56% of cases tested positive for *SS18* gene rearrangements via FISH, lower than the 90% reported in other studies [20]. Notably, RT-PCR confirmed the presence of *SS18-SSX1* transcripts in 60% of cases and *SS18-SSX2* in 32%. These findings are consistent with global literature; for example, a study by [21] found 62% positivity for *SS18-SSX1* in their cohort. The high concordance between FISH and RT-PCR underscores the reliability of molecular confirmation for *SS18-SSX* fusion and highlights the necessity of integrating molecular techniques for more precise diagnosis in synovial sarcoma.

Although some cases in our series were categorized as fusion-negative and considered undifferentiated sarcomas, we acknowledge that additional fusion partners or alternative fusion isoforms may have escaped detection with the methods applied in this study. Previous reports have described novel or variant fusions in synovial sarcoma [10,22,23,24], highlighting the possibility that some of our fusion-negative cases may harbor yet unidentified rearrangements.

The heatmaps of EMA and CK expression provided essential insights into how tumor morphology and sex influence marker expression in synovial sarcoma. In the EMA heatmap, female patients with spindle-shaped and round cell morphologies consistently exhibited higher positivity compared to male patients, suggesting a potential sex-specific interaction that could affect the biological behavior of these tumors. In contrast, the consistently high EMA positivity in epithelial tumors across both sexes’ highlights tumor morphology as a stronger determinant of EMA expression, indicating that epithelial differentiation may override sex-related variability.

Similarly, CK expression patterns demonstrated a more uniform distribution between sexes for spindle-shaped tumors, suggesting that CK may not be sensitive to sex-related differences in this morphological subtype. The low CK expression in both sexes for round cell tumors points to a distinct molecular profile, possibly indicating a less differentiated state compared to epithelial counterparts. Epithelial tumors showed consistently high levels of both CK and EMA expression, reinforcing their association with a more differentiated tumor phenotype.

Furthermore, the MDS plots revealed that neither tumor grade nor sex significantly differentiates fusion type in synovial sarcoma patients. The dispersion of *SYT-SSX1* and *SYT-SSX2* across grades and the absence of distinct clustering by sex suggest that these factors do not exhibit strong interdependence. Notably, the t-test results confirmed these observations, revealing no significant differences in tumor grades between fusion types (*p*-values: 0.92 for *SYT-SSX1* and 0.68 for *SYT-SSX2*) and demonstrating that sex does not influence fusion type distribution (*p*-value = 1.00). This finding contrasts with some literature suggesting a more pronounced influence of sex on tumor characteristics [19].

While our findings provide valuable insights into the clinicopathological characteristics of synovial sarcoma, they also underscore the complexity of the disease. The observed sex-related differences in EMA expression for spindle-shaped and round cell tumors warrant further investigation to understand their clinical implications, such as differing responses to treatment or prognostic outcomes between male and female patients. Furthermore, we found no significant associations between *SYT-SSX* fusion types and clinical features like gender, tumor diameter, or histologic type, which may stem from the limited sample size, emphasizing the need for larger studies.

Future research should focus on characterizing the genetic landscape of Moroccan patients with synovial sarcoma to yield a comprehensive understanding of the genetic and epigenetic factors driving tumor progression and therapeutic resistance. Developing targeted therapies aimed at inhibiting *SS18-SSX* fusion proteins is crucial for improving patient outcomes, especially amidst ongoing debates regarding the efficacy of conventional chemotherapy.

This study is limited by the rarity of the disease and the small number of patients included, which may affect the generalizability of our findings. While the techniques described in this study are increasingly applied in specialized laboratories, they are not yet considered standard practice in all centers.

The economic implications and costs of these analyses should also be considered, as they may limit accessibility in some healthcare settings.

## 5. Conclusions

This study provides valuable insights into the clinicopathological and molecular characteristics of synovial sarcoma in a Moroccan cohort. *SYT-SSX* fusion types did not show significant associations with sex, tumor grade, or location, suggesting that multiple factors influence disease progression. Immunohistochemical analysis confirmed strong EMA and CK expression in specific morphologies, but statistical analysis found no clear clinical patterns. These findings highlight the need for larger studies and molecular profiling to improve diagnosis and treatment strategies for synovial sarcoma, particularly in developing targeted therapies.

## Data Availability

The data are not publicly available due to patient confidentiality and institutional regulations. Data may be shared by the corresponding author upon reasonable request and with appropriate ethical approvals.

## Supplementary Materials

The following supporting information can be downloaded at: https://www.mdpi.com/article/10.3390/jpm15100455/s1, Figure S1: Example of β-2M gene amplification; Figure S2: RT-PCR and nucleotide sequence analysis of the *SS18-SSX* fusion gene; Figure S3: Electropherogram of the amplified *SYT-SSX1* gene in patient number 5; Table S1: Clinical and Histopathological Characteristics of Synovial Sarcoma Patients in our cohort.

## Abbreviations

The following abbreviations are used in this manuscript:

β-2M	Beta-2 Microglobulin
BLAST	Basic Local Alignment Search Tool
CK	Cytokeratin
cDNA	Complementary DNA
DAB	3,3′-diaminobenzidine tetrahydrochloride
DNA	Deoxyribonucleic acid
EMA	epithelial membrane antigen
FFPE	formalin-fixed, paraffin-embedded
FISH	Fluorescence In Situ Hybridization
FNCLCC	Fédération Nationale des Centres de Lutte contre le Cancer
H&E	hematoxylin and eosin
HIER	Heat-induced epitope retrieval
IHC	Immunohistochemistry
LSI	Locus-Specific Identifier
OR	Odds ratio
MDS	Multidimensional Scaling
PCR	Polymerase Chain Reaction
RNA	Ribonucleic acid
RT-PCR	Reverse transcription–polymerase chain reaction
SS	Synovial sarcoma
SYT	Synovial Sarcoma Translocation
