# Supplementary material for: Molecular Profiling of SYT-SSX Fusion Transcripts for Enhanced Diagnosis of Synovial Sarcomas
Source: J Pers Med. 2025 Sep 29;15(10):455. doi: 10.3390/jpm15100455 (PMC12565586; doi:10.3390/jpm15100455)
Supplement: Supplementary file 1 [file jpm-15-00455-s001.zip › jpm-3813333-supplementary.pdf]

Supplementary Figure S1 : Example of *β-2M* gene amplification to test mRNA quality in some cases of synovial sarcomas on a 2% agarose gel. [S1-S13] patients, R2, R8, and R9 are positive controls.

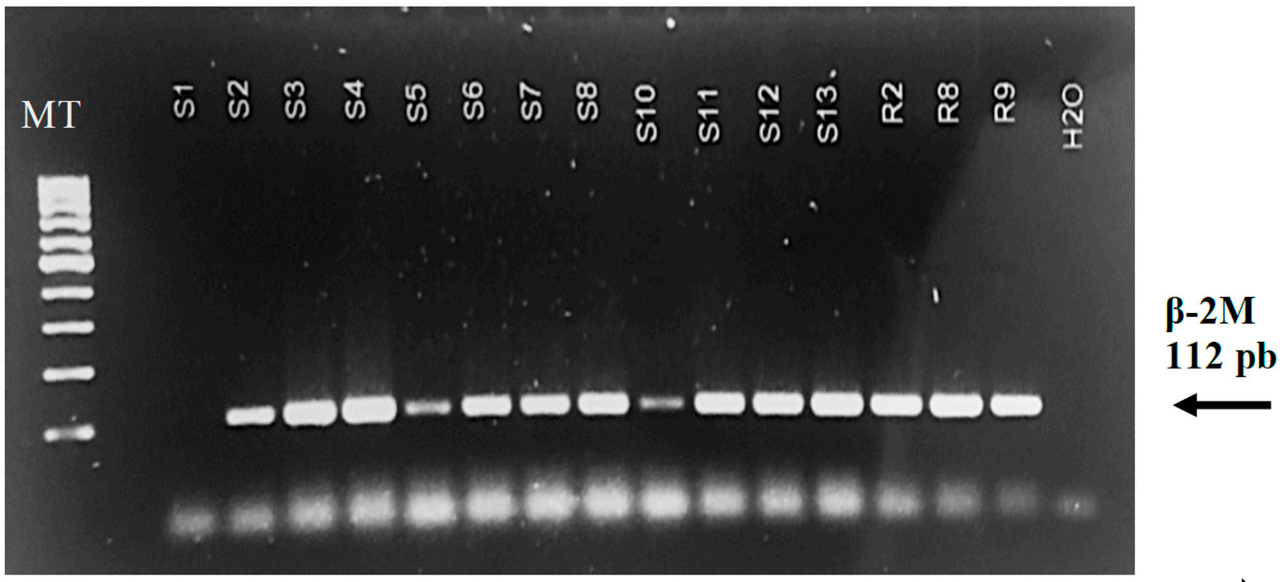

Supplementary Figure S2: RT-PCR and nucleotide sequence analysis of the *SS18-SSX* fusion gene. RT-PCR products were analysed via gel electrophoresis. The whole gel was represented, and its margin was cropped. Lane MT, molecular size; [S1-S13] patients, R2 and R8 are positive controls for *SYT-SSX1*, and R9 is a positive control for *SYT-SSX2*. The *SYT-SSX1* (109 pb) and *SYT-SSX2* (109pb) fusion transcripts in synovial sarcoma cases were detected on a 2% agarose gel.

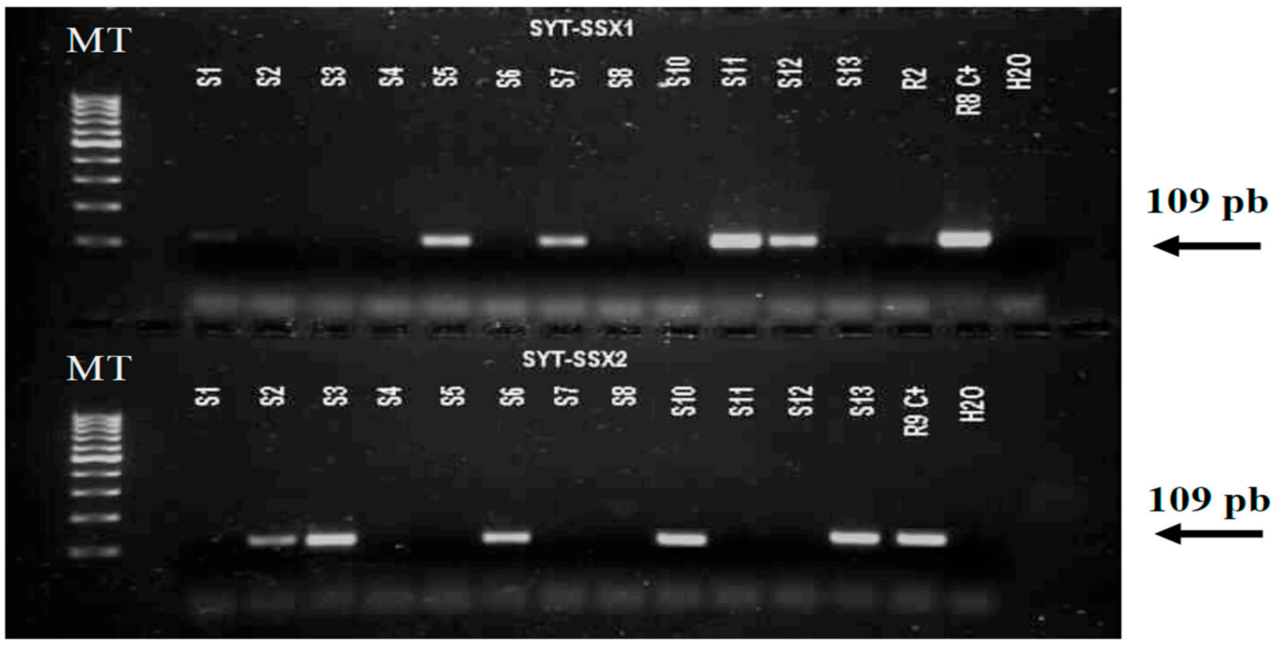

Supplementary Figure S3 : Electropherogram of the amplified *SYT-SSX1* gene in patient number 5. The sequence is aligned with Homo sapiens Synovial Sarcoma, X break point 1 (SSX1). This electropherogram shows the amplified sequence of the SYT-SSX1 fusion gene. The fusion site (arrowhead) for the *SS18-SSX1* fusion transcript is involved in exon 10 of the *SS18* gene (codon 410) and exon 6 of the *SSX1* gene (codon 111). The sequence is aligned with Homo sapiens Synovial Sarcoma X breakpoint 1 (SSX1). The distinct peaks confirm the successful amplification and sequencing of the *SYT-SSX1* transcript, which is critical for confirming the molecular diagnosis of synovial sarcoma.

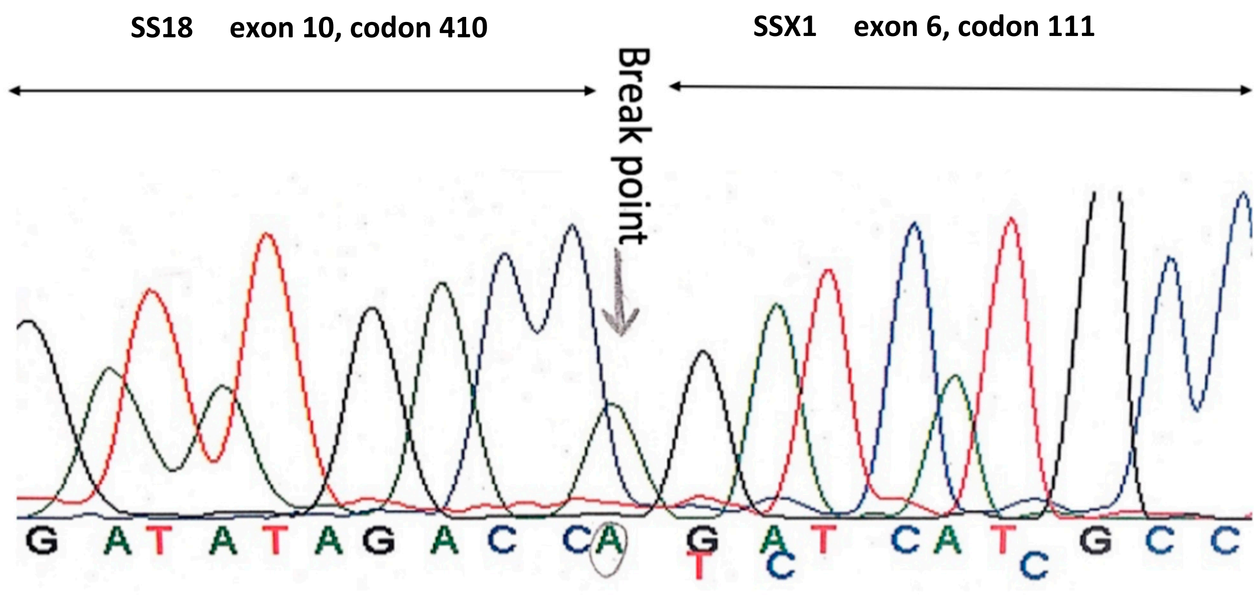

Table S1: Clinical and Histopathological Characteristics of Synovial Sarcoma Patients in our cohort. F female, M male, FNCLCC grading system: Fédération Nationale des Centres de Lutte contre le Cancer, IHC Immunohistochemistry, Ck cytokeratin, EMA epithelial membrane antigen.

Chracteristics	Cases (N=48)	Pourcentage
**Age**		
[11, 19]	9	18,75%
[20, 29]	12	25%
[30, 39]	7	14,6%
[40, 49]	6	12,5%
[50, 59]	7	14,6%
[60, 69]	6	12,5%
[70, 77]	1	2,05%
**Sex**		
F	21	43,75%
M	27	56,25%
**Location of the tumor**		
Lower limbs	29	60,5%
Upper limbs	6	12,5%
Central axis	13	27%
**Histomorphology**		
Fusiform	30	62,5%
Round	12	25%
Epithelial component	6	12,5%
**FNCLCC grading**		
2	28	58,3%
3	20	41,7%
**IHC**		
Ck+	21	56,25%
EMA+	35	72,9%
